# Facile one-pot synthesis and in silico study of new heterocyclic scaffolds with 4-pyridyl moiety: Mechanistic insights and X-ray crystallographic elucidation

**DOI:** 10.1016/j.heliyon.2024.e29221

**Published:** 2024-04-03

**Authors:** Fathy M. Abdelrazek, Magdi E.A. Zaki, Sami A. Al-Hussain, Basant Farag, Ali M. Hebishy, Mohamed S. Abdelfattah, Safaa M. Hassan, Ahmed F. El-Farargy, Lyuba Iovkova, David Mross, Sobhi M. Gomha

**Affiliations:** a Chemistry Department, Faculty of Science, Cairo University, Giza, 12613, Egypt; b Department of Chemistry, Faculty of Science, Imam Mohammed Ibn Saud Islamic University (IMSIU), Riyadh, 11623, Saudi Arabia; c Department of Chemistry, Faculty of Science, Zagazig University, Zagazig, 44519, Egypt; d Chemistry Department, Faculty of Science, Helwan University, Helwan, 11795, Cairo, Egypt; e Fakultät für Chemie und Chemische Biologie, TU Dortmund, Dortmund, 44227, Germany; f Department of Chemistry, Faculty of Science, Islamic University of Madinah, Madinah, 42351, Saudi Arabia

**Keywords:** 4-Acetyl pyridine, Chromenes, X-ray crystallography, Cyclization, Molecular docking studies, And in silico ADMET

## Abstract

4-Acetylpyridine **1** and malononitrile **2** were allowed to react in a 3MCRs with dimedone **3a** or cyclohexa-1,3-dione **3b** under reflux to afford 4-methyl-4-(pyridin-4-yl)-5,6,7,8-tetrahydro-4*H*-chromene derivatives **4a,b** respectively. The mechanism of the reaction has been studied and the structures elucidated by analytical, spectral as well as X-ray crystallographic data. Heterocyclic compounds find widespread application in pharmaceutical and agrochemical products. Docking analyses were performed on the synthesized compounds to assess their binding modes with various amino acids of the target protein tubulin (PDB Code - 1SA0). The results indicated promising binding scores for compounds **4a** and **4b**, suggesting a strong affinity for the tubulin binding site. Finally, ADMET for the synthesized compounds **4a, 4b, 5, 8a** and **8b** were carried out. The drug likeness and pharmacokinetic properties of the prepared compounds were also evaluated. Notably, all of the novel compounds adhered to Lipinski's rule (Ro5) without any violations.

## Abbreviations

EtOH: ethanol
MeOH: methanol
3MCRs: three multicomponent reactions
GDP: Guanosine diphosphate; Lipinski's rule (Ro5)
ADMET: absorption, distribution, metabolism, excretion, and toxicity
NaOEt: sodium methoxide
RMSD: root mean square deviation
HA: hydrogen bond acceptors
D_2_O: deuterium oxide
ppm: part per million
s: singlet
d: doublet
t: triplet
q: quartet

## 1 Introduction

Functionalized pyridines exhibit a wide range of pharmaceutical properties, including antiviral [1], antimicrobial [2,3], anticonvulsant [4], antifungal, and antimycobacterial [5], as well as anti-HIV [6] and anti-tumor [[7], [8], [9], [10], [11]] activities. Chromenes also exhibit diverse biological activities such as molluscicidal [12], and potent anti-leishmanial agents [13]. They also possess antipyretic, analgesic, anti-inflammatory and antioxidant [14] as well as anticancer activity [14,15]. The biological activities of chromene derivatives [[16], [17], [18], [19], [20]] have attracted significant attention, leading to numerous reports on their syntheses using various methods, including microwave solvent-free enhanced synthesis [21], ultrasonic synthesis [22,23], and the utilization of benign, ecofriendly, and green catalysts [[24], [25], [26], [27]].

**Fig. 1:**
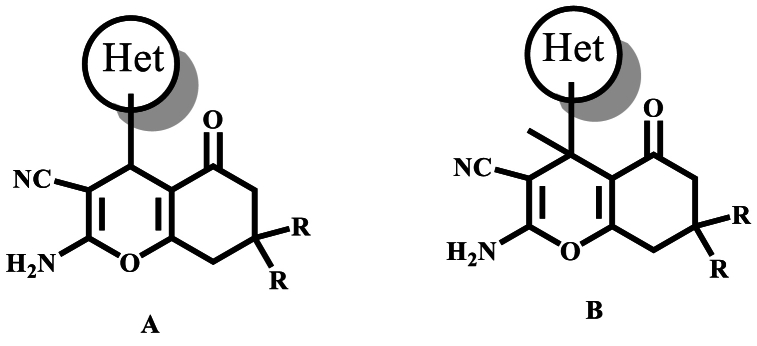
Structures of 4-hetaryl chromenes **4a** and 4-hetaryl-4-methyl chromenes **4b**.

However, while the majority of these studies have focused on the synthesis of 4-hetaryl derivatives (like A; Fig. 1), there are only a few reports describing the synthesis of 4,4-dimethylchromene and 4-methyl-4-(methoxymethyl)-chromene [28], spiroindenoquinoxaline chromene and pyrazole [29], 4,4-disubstituted pyrano [2,3-c]pyrazoles with different substituents, and spiro-derivatives [30,31]. Notably, except for one publication from our laboratory [32], no other report has described the synthesis of 4-hetaryl-4-methyl derivatives (like B; Fig. 1). Therefore, we are intrigued by the opportunity to explore the synthesis of these seldom known compounds.

N-Heterocyclic skeletons have found extensive use in various therapeutic applications due to the nitrogen atom's capacity to readily form hydrogen bonds with biological targets [6,7,11]. This property has made them a fundamental basis for numerous potential drug candidates [33]. Recent research, as discussed in article [34], has been dedicated to exploring the biological applications of nitrogen-containing molecules. Among these, those featuring a nitrogen atom in six-membered heterocyclic motifs, especially 4-pyridyl, have gained significant recognition for their crucial role in therapeutics [6,7]. Further molecular docking studies were conducted using the binding protein at the tubulin interface (PDB ID: 1SA0) and good docking scores were recorded [35,36]. The 4-pyridyl moiety has attracted substantial interest in chemical, medical, and pharmaceutical research due to its ability to form hydrogen bonds with the receptor site [37,38], offering promise for the development of innovative drugs [2,6]. The choice to incorporate hetero-atom in these molecules is not arbitrary but rather based on their specific physicochemical properties, with a focus on optimizing ADMET (absorption, distribution, metabolism, excretion, and toxicity) for the main therapeutic agents [39,40]. Additionally, the novel derivatives underwent drug-likeness analysis to assess their potential suitability as drug candidates [39,40].

**Scheme 1:**
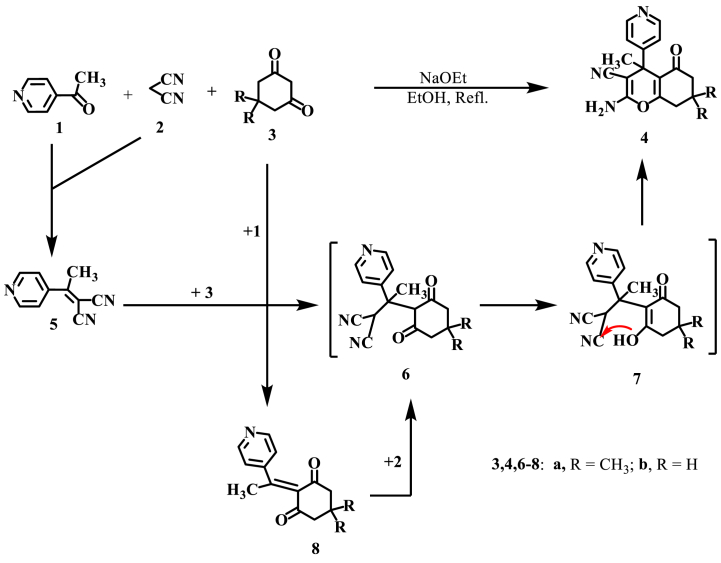
Synthesis of compounds **4a** and **4b**.

In the context of our program aiming at the synthesis of some novel heterocyclic scaffolds of anticipated biological activity; we thought that the combination of a pyridine moiety with a chromene moiety in one entity may lead to more pronounced biological activity due to the synergistic effects of both rings. 4-Acetyl pyridine **1** seemed appropriate candidates to fulfill this objective via its reaction with malononitrile **2** and cyclic β-diketones namely dimedone **3a** (R=CH_3_) and cyclohexa-1,3-dione **3b** (R=H) (*cf*. Scheme 1).

## 2 Results and discussion

Thus, 4-acetylpyridine **1** and malononitrile **2** were allowed to react in a 3MCR with dimedone **3a** or cyclohexa-1,3-dione **3b** in refluxing EtOH catalyzed by few drops of NaOEt to afford quantitative yields of analytically pure products with mp's. 235 and 223 °C for which structures **4a** and **4b** are assigned respectively (Scheme 1).

This reaction likely starts with the condensation of 4-acetylpyridine **1** and malononitrile **2**, resulting in the formation of an aralkylidene malononitrile derivative **5**. Subsequently, this derivative undergoes a Michael-type addition with the active methylene of either **3a** or **3b**, leading to the formation of acyclic adducts (**6a** and **6b**). These adduct then enolize to form **7a** and **7b**, which subsequently undergo *in situ* cyclization to yield **4a** and **4b**, respectively.

Alternatively, this reaction involves initial condensation of the active methylene of **3a** and **3b** with 4-acetylpyridine **1** to afford the condensation products **8a** and **8b** which add malononitrile **2** to the activated double bond to give the same acyclic intermediates (**6** → **7**), which cyclize to furnish **4a** and **4b**, respectively (Scheme 1). To validate these suggestions, the diketones **3a** and **3b** underwent reactions with the following compounds: (a) Aralkylidene malononitrile derivatives **5**, which were prepared separately by condensation of pyridyl methyl ketone **1** with malononitrile **2**. (b) Pyridyl methyl ketone **1** itself, resulting in the formation of aralkylidene derivatives **8a** and **8b.**

The aralkylidene derivatives **8a** and **8b** were further reacted with malononitrile **2** to produce identical acyclic intermediates (**6** → **7**). These intermediates subsequently underwent cyclization to yield the products **4a** and **4b**, respectively (refer to Scheme 1).The products isolated from both routes (a) and (b) were found to be completely identical in all respects with compounds **4a** and **4b** presumably via the same intermediates (**6** → **7**) respectively. The mass spectra of these two obtained products showed molecular ion peaks at *m*/*z* = 309.36 and 281.31, respectively.

The IR spectra of both **4a** and **4b** showed absorption peaks at υ_max_ ~ 3325 & 3212, 2194, 1677 cm^−1^ assignable to the amino, cyano and carbonyl groups, respectively [41,42]. The ^1^H NMR spectrum of **4a** revealed two equivalent methyl signal at *δ*_H_ = 1.0 ppm (s, 6H) and one methyl signal at *δ*_H_ = 1.55 ppm (s, 3H), a singlet signal (s, 2H, D_2_O exchangeable) assignable the amino group a *δ*_H_ = 6.9 ppm, beside the other signals of the pyridine and chromene rings (see Experimental). The ^1^H NMR spectrum of **4b** revealed a similar pattern except only one methyl singlet at *δ*_H_ = 1.73 ppm and three methylene multiplets at *δ*_H_ = 1.85–1.97, 2.16–2.22, and 2.44–2.54 ppm [41,42]. ^13^C NMR spectrum of **4a** revealed 15 signals two of them are extremely up field quartets at *δ*_C_ = 25.9 (q); 27.5 (q) with relative intensity 1:2 denoting to three methyl groups and a one downfield carbonyl singlet at *δ*_C_ = 198.9 (s) due to the carbonyl group, beside the other signals at their expected positions (*cf*. Scheme 1 and Experimental section). ^13^C NMR spectrum of **4b** revealed a similar pattern except only 14 signals; one methyl quartet at *δ*_C_ = 25.92 ppm and three methylene triplets at *δ*_C_ = 21.15, 29.3 and 37.05 ppm beside the other signals at their expected positions (*cf*. Scheme 1 and Experimental section).

**Fig. 2:**
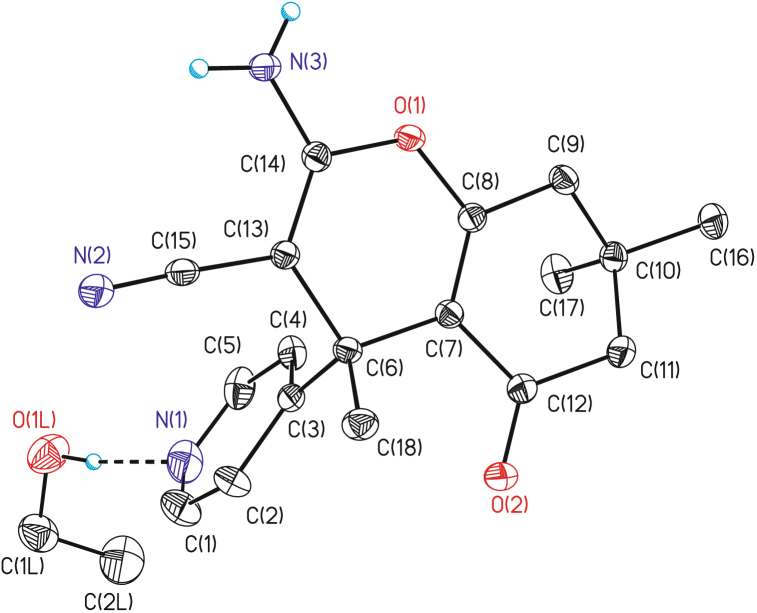
The 30% probability ellipsoids of the depicted atoms and the atom numbering scheme of compound **4a** are presented using ORTEP. Non-bridged hydrogen atoms have been omitted for clarity.

The X-ray crystallography of compound **4a** gives decisive evidence of its structure as shown in Fig. 2 and experimental section [43]. The molecule is shown to be hydrogen bonded with EtOH molecule through the pyridine nitrogen.

X-ray crystallographic data: colorless crystals, C_20_H_25_N_3_O_3_ (Mwt = 355.43 g/mol) [C_18_H_19_N_3_O_2_ (309.36) + EtOH (46.07)], audit creation method and version "SHELXL-2018/3". Crystal dimensions = 0.132 * 0.253* 0.418 mm. Molecules per unit cell Z = 2, Dcalcd = 1.251 g/cm^3^. F(000) = 380, crystal system: triclinic, space group P-1 (no. 2), cell constants with standard deviations: a = 8.3531(5) Ǻ, b = 9.4448 (5) Ǻ, c = 12.8758(8) Ǻ, α[°] = 88.808(2), β[°] = 73.197(2), γ[°] = 75.084(2); Cell volume 938.10(10) Ǻ^3^. The data were obtained using a Bruker APEX-II CCD at a temperature of T [°C] = 100.0 (2) K, with a graphite monochromator using Mo Kα radiation (λ = 0.71073 Å). The CCD data collection and SADABS absorption correction method were employed. The absorption coefficient was μ = 0.086 mm^−1^, and the maximum resolution was 2θ max = 30.100. The absorption correction ranged from a minimum of 0.965 to a maximum of 0.989. A total of 9925 reflections were measured in the cell, out of which 54104 were independent reflections, and 5521 were observed reflections. The Rint value was found to be 0.0759.

The intensity data for the colorless crystals of compound **4a** were obtained using a Bruker APEX-II CCD diffractometer with Mo-Kα radiation at 100 (2). The molecular structures were solved employing direct methods with SHELXT-2014/7, and refinements against F2 were performed using SHELXL-2018/3, as described by G. M. Sheldrick [44]. The C–H hydrogen atoms were placed with idealized geometry and refined using a riding model.

Supplementary crystallographic data for this paper are available under the identifier CCDC 2241780 (**4a**). The data can be obtained without any charge from The Cambridge Crystallographic Data Centre website at www.ccdc.cam.ac.uk/data_request/cif. Decimal rounding of numerical parameters and su values was performed following the guidelines of IUCr [45]. All figures in this paper were created using ORTEP III [46,47].

### 2.1 Molecular docking studies

**Fig. 3:**
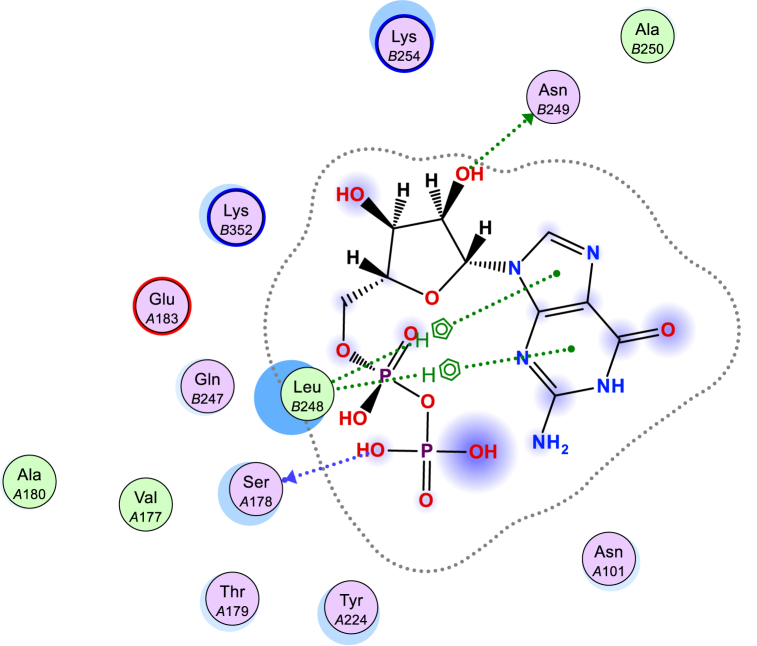
Interacting amino acids at the active site of the protein with Guanosine diphosphate (GDP).

**Fig. 4:**
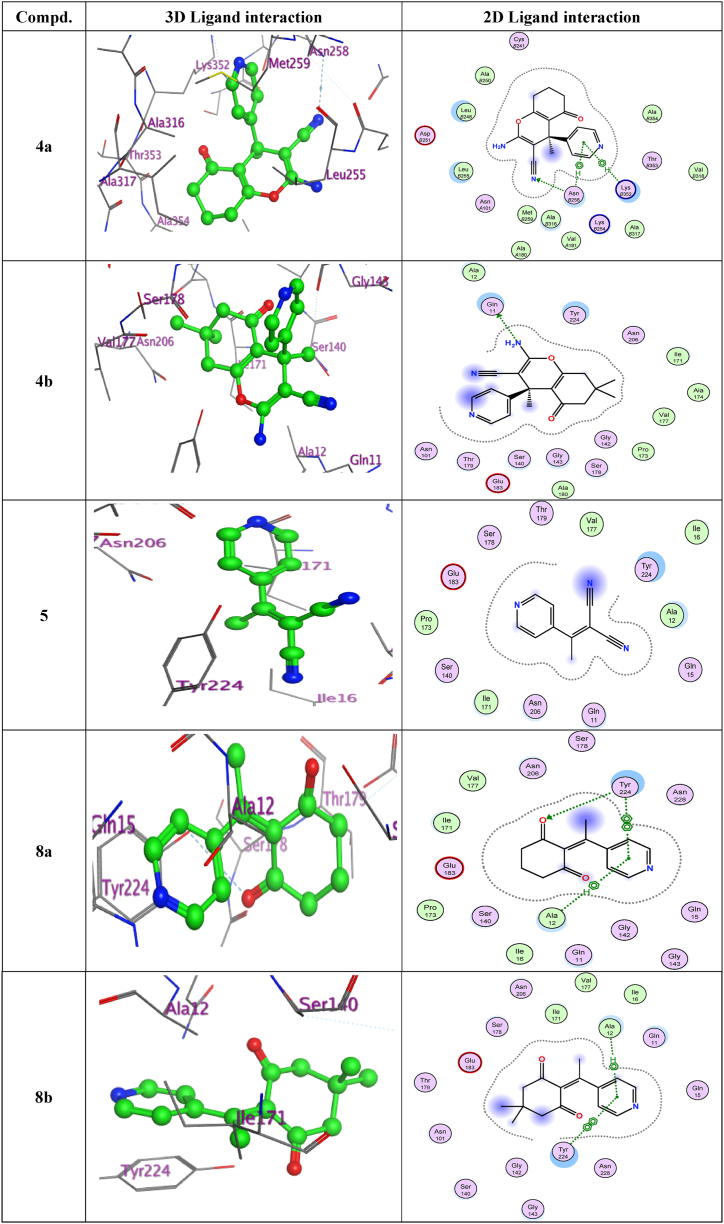
Analysis of 3D and 2D ligand interactions within the binding site of 1SA0 was conducted for a series of synthesized compounds, namely **4a, 4b, 5, 8a,** and **8b**.

The interactions between the designed compounds and the targeted protein were examined through molecular docking studies [48]. The ligand structures were docked at binding site of PDB ID-1SA0 was found to be stabilized in the cavity via hydrogen bonding interactions and arene interactions [49,50]. Target protein give the best conformation of the ligand's evaluation [51]. The evaluation of docked molecules involved assessing their binding affinity scores and the identification of noteworthy hydrogen bonds, arene interactions, and RMSD [root mean square deviation] [52] (as shown in Table 1). Target enzyme is used since it includes the conserved region as well as the active site [51]. The crystal structure of tubulin with GDP [53] provides critical insights for designing specific drugs, highlighting the significance of studying tubulin in advancing drug discovery [54]. The results of the docking were compared with those of GDP [55], which also showed interactions with specific protein residues (as illustrated in Fig. 3) [56]. In Tables 1 and it is evident that the test compound **4a** exhibited an H‐bond acceptor interaction with Asn258 through the nitrogen atom of its cyano group. Additionally, the pyridyl part of **4a** formed two pi-H interactions, one with Asn258 and another with Lys352. The 3D model of compound **4b** demonstrated a hydrogen bond donor involving its amino group's nitrogen and Gln11. Furthermore, compounds **8a** and **8b** showed interesting interactions with the protein. The pyridyl moiety of both compounds engaged in pi-H interaction with Ala12. Also, these two compounds formed a pi-pi interaction with Tyr224. Additionally in compound **8a** exhibited a hydrogen bond acceptor interaction between its oxygen atom and Tyr224 (Fig. 4).

**Table 1:** The docking score, number of hydrogen bonds, number of arene interaction, and RMSD were determined for the synthesized compounds **4a, 4b, 5, 8a,** and **8b** when they were compared to GDP, using the 1SA0 receptor.

Cpd. NO.	Docking score (kcal/mol)	NO. of hydrogen bonding	NO. of arene interaction	RMSD kcal·mol^−1^Å^−1^
**4a**	−6.6	1 (Asn258)	1 (pi-*H*) [Asn258] 1 (pi-*H*) [Lys352]	0.9
**4b**	−6.1	1 (Gln11)	–	1.8
**5**	−5.4	–	–	1.3
**8a**	−5.9	1 (Tyr224)	1 (pi-*H*) [Ala12] 1 (pi-pi) [Tyr224]	1.3
**8b**	−5.9	–	1 (pi-*H*) [Ala12] 1 (pi-pi) [Tyr224]	1.3
**GDP**	−6.0	1 (Ser178) 1 (Asn249)	2 (pi-*H*) [Leu248]	1.3

### 2.2 ADME property study

The Swiss ADME tool was used to evaluate the Absorption, Distribution, Metabolism, and Excretion (ADME) properties of the drugs [57]. This tool predicts the physicochemical and pharmaceutical properties of organic compounds [57,58]. To evaluate certain physicochemical descriptors for derivatives **4a**, **4b**, **5**, **8a**, and **8b** in comparison to GDP, a reference drug, researchers utilized the Swiss ADME web tool (http://swissadme.ch/index.php, accessed on July 26, 2023), as described in a previous study [59]. Table 2 presents the predicted descriptors, classified into molecular properties, pharmacokinetics, drug-likeness, and medicinal chemistry. Compounds that exhibit drug-like properties and have been well-studied make excellent candidates for further therapeutic development. The critical physicochemical characteristics assessed included molecular weight, lipophilicity, the number of hydrogen bond donors (HBD) and acceptors (HBA), rotatable bonds (ROT), and polar surface area (PSA). The results, summarized in Table 2, showed that the molecular weights of the proposed compounds ranged from 169.18 to 309.86, indicating a diverse representation of physicochemical characteristics data. The iLogP values fell between 1.43 and 2.15, reflecting their moderate lipophilicity. The number of hydrogen bond acceptors (HA) ranged from 3 to 4, and all the compounds had zero to one hydrogen bond donors. Additionally, all synthesized compounds possessed one rotatable bond, except for the molecule GDP (GUANOSINE-5′-DIPHOSPHATE), which had 6 rotatable bonds. Notably, all of the novel compounds adhered to Lipinski's rule (Ro5) without any violations. The physicochemical properties of substances with decreased molecular weight and lipophilicity play a significant role in improving paracellular and transcellular absorption, enhancing renal excretion, and causing no toxicity [60,61]. Regarding pharmacokinetic prediction, some of them (**4a**, **4b**, and GDP) did not pass the blood-brain barrier (BBB). GDP showed low gastrointestinal tract permeability, while the others exhibited high permeability. With the exception of **4a** and **4b**, the derivatives were not substrates for P-gp. A compound that acts as a P-gp substrate can reduce drug accumulation and commonly promote resistance in multidrug-resistant cells. Furthermore, these derivatives showed a permeability coefficient (LogKp = −6.97 to −6.02 cm/s) compared to GDP, which demonstrated a low chance of crossing the skin with a predicted LogKp = −12.29 cm/s. Additionally, neither the derivatives nor GDP contained pan assay interference compounds (PAINs) in their structures. All the synthesized derivatives complied with the Lipinski and Veber rules, demonstrating their potential as promising drug candidates. The adherence to specific criteria, such as the "rule of 5" (Ro5) and Veber rule, helps determine whether a molecule can be considered drug-like and have superior oral bioavailability [62]. Moreover, the derivatives exhibited moderate synthetic accessibility scores (1.99–4.02), while GDP showed an easy synthetic accessibility score of 4.68. For all ligands in this investigation to function as effective oral medications, the ideal value for a neutral molecule was determined to be a bioavailability score (ABS). The bioavailability scores for both tested compounds were 0.55 and 0.56, respectively.

**Table 2:** An analysis of molecular properties, pharmacokinetics, drug-likeness, and medicinal chemistry was conducted for derivatives **4a, 4b, 5, 8a,** and **8b** in comparison with GDP.

Test items	4a	4b	5	8a	8b	GDP
Molecular properties						
PSA (^o^A^2^)	89.00	89.00	60.47	47.03	47.03	272.19
M. Wt.	281.31	309.86	169.18	215.25	243.30	243.20
HBA	4	4	3	3	3	13
HBD	1	1	0	0	0	7
NRB	1	1	1	1	1	6
iLogp	1.81	2.15	1.43	1.69	2.06	−1.62
**Pharmacokinetics**						
BBB permeant	No	No	Yes	Yes	Yes	No
GI absorption	High	High	High	High	High	Low
P-gp substrate	Yes	Yes	No	No	No	No
Skin permeation (Log Kp) cm/s	−6.97	−6.55	−6.23	−6.43	−6.02	−12.29
**Drug likeness and Medicinal Chemistry**						
PAINS	0	0	0	0	0	0
Veber Rule (violation)	Yes	Yes	Yes	Yes	Yes	No (1)
Lipinski Rule (violation)	Yes (0)	Yes (0)	Yes (0)	Yes (0)	Yes (0)	No (2)
Synthetic accessibility	3.81	4.02	1.99	2.27	2.50	4.68
Bioavailability Score	0.56	0.56	0.55	0.55	0.55	0.11

These characteristics are crucial for the development of a molecule with improved absorption, distribution, metabolism, and excretion (ADME), making it a potential therapeutic candidate [[63], [64], [65]].

### 2.3 In silico pharmacokinetic profile (ADMET)

The pharmacokinetic profile of synthesized compounds **4a, 4b, 5, 8a** and **8b** was expaned using pkCSM [66]. It was observed that all the compounds were exhibited less water solubility (−4.05: −1.84 log mol/L). The Caco2 permeability (log Papp in 10^−6^ cm/s) was found nearly 1 and the percentage of intestinal absorption was ranging from 96.69 to 98.55. The volume of distribution (log L/kg) for the compounds **4a, 4b, 5, 8a** and **8b** was −0.11, −0.01, −0.18, −0.12, and −0.05 respectively and the fraction unbound was 0.35, 0.27, 0.45, 0.46, and 0.37 respectively. None of the compounds was exhibited the CNS permeability. In the metabolism part, compound **4b** inhibited CYP3A4. The compound **5** was also inhibited CYP1A2. The excretion of the compounds (**4a**, **4b**, **5**, **8a** and **8b**) measured with log value of ml/min/kg and the values are 0.53, 0.36, −0.79, 0.52, and −0.18 respectively. All pervious detailed dates were shown in Table 3.

**Table 3:** Results of ADME properties of compounds **4a, 4b, 5, 8a** and **8b**.

Entry	Absorption			Distribution					
	Log S (log mol/L)	Caco2 perm. (log < Papp in 10^−6^ cm/s)	Int. abs.(% abs.)	VDss (Log L/kg)	Fract. unb. (Fu)	BBB perm. (log BB)	CNS perm. (log PS)	Metabolism	Excretion Log(ml/min/kg)
**4a**	−3.44	1.10	96.69	−0.11	0.35	−0.28	−2.89	–	0.53
**4b**	−4.05	1.15	96.85	−0.01	0.27	−0.26	−2.85	CYP3A4 substrate	0.36
**5**	−1.84	1.30	97.27	−0.18	0.45	0.06	−2.75	CYP1A2 inhibitior	−0.79
**8a**	−2.14	1.32	98.55	−0.12	0.46	0.37	−2.81	–	0.52
**8b**	−2.95	1.36	98.37	−0.05	0.37	0.38	−2.74	–	−0.18

Later, the results of toxicity prediction (Table 4) revealed that all compounds have not AMES toxicity. All the five compounds were inert towards hERG I and hERG II inhibition, but some of them (**4a** and **4b**) shown hepatotoxicity. All the synthesized (except **8a**) compounds were inert towards Skin Sensitization. The maximum tolerated dose (human; expressed in log value of mg/kg/day) of compounds (**4a, 4b, 5, 8a** and **8b**) was o.27, 0.21, 0.83, 0.64, and 0.56 respectively.

**Table 4:** Toxicity prediction of compounds **4a, 4b, 5, 8a** and **8b**.

Entry	AMES toxicity	hERG I inhibitor	hERG II inhibitor	Hepatotoxicity	Skin Sensitization	Max. tolerated dose (human); log (mg/kg/day
**4a**	No	No	No	Yes	No	o.27
**4b**	No	No	No	Yes	No	0.21
**5**	No	No	No	No	No	0.83
**8a**	No	No	No	No	Yes	0.64
**8b**	No	No	No	No	No	0.56

## 3 Experimental

Melting points were determined using an Electrothermal 9100 apparatus at Kleinfeld in Gehrden, Germany. The ^1^H NMR and ^13^C NMR spectra were obtained using a Bruker AC 300 P (^1^H NMR: 300 MHz, ^13^C NMR: 75 MHz; Bruker, Rheinstetten, Germany) in DMSO‑*d*_6_, with TMS as the internal reference. The chemical shifts are expressed in *δ* (ppm) values. The ^13^C multiplicities were determined using DEPT and off-resonance pulse sequences. X-ray data [27,28] were collected with a Bruker Nonius Kappa diffractometer at Bruker in Rheinstetten, Germany, and corrected using SADABS factors and empirical absorption. The FTIR spectra (KBr) were recorded on a Nicolet 205 spectrophotometer manufactured by Nicolet in Madison, WI, USA. The graphic representation of the structure utilized the program SCHAKAL 99 [29]. X-ray crystallography was conducted in the Microanalytical laboratory of the Fakultät für Chemie und Chemische Biologie at TU Dortmund, Germany. The spectral and elemental analyses were carried out in the Microanalytical Center at Cairo University in Cairo, Egypt.

### 3.1 Synthesis of 4-methyl-4-(pyridin-4-yl)-5,6,7,8-tetrahydro-4*H*-chromene derivatives 4a and 4b

A mixture containing 1.21 g (10 mmol) of 4-acetylpyridine **1**, 0.66 g (10 mmol) of malononitrile **2**, and the appropriate cyclic β-diketone (1.4 g (10 mmol) of dimedone **3a** or 1.12g (10 mmol) of cyclohexane-1,3-dione **3b**) was dissolved in 25 mL of absolute EtOH. The contents were heated until complete dissolution. Next, a few drops of freshly prepared NaOEt were added to the mixture. The reaction mixture was then refluxed for an additional 1 h, monitored by TLC (using MeOH/hexane 1:9 as the solvent). Afterward, it was left to cool overnight. The formed crystalline solids were filtered, washed with cold EtOH, dried, and finally recrystallized from EtOH to obtain compounds **4a** and **4b**, respectively. The overall yield of the reaction was generally higher when using dimedone compared to cyclohexane-1,3-dione.

**2-Amino-4,7,7-trimethyl-5-oxo-4-(pyridin-4-yl)-5,6,7,8-tetrahydro-4*H*-chromene-3-carbonitrile 4a:** Colorless crystals (EtOH), yield (2.78 g, 90%), mp. 235 °C. IR υ_max_ = 3325 & 3212 (NH_2_), 2194 (CN), 1677 (C=O) cm^−1^. ^1^H NMR: *δ*_H_ = 1.0 (s, 6H, 2CH_3_), 1.55 (s, 3H, CH_3_), 2.1 (dd, 2H, CH_2_), 2.6 (dd, 2H, CH_2_), 6.9 (s, 2H D_2_O exchangeable, NH_2_), 7.3 (d, *J* = 5.1 Hz, 2H, pyridine-H_3_, H_5_), 8.45 (d, *J* = 5.1 Hz, 2H, pyridine-H_2_, H_6_) ppm. ^13^C NMR: *δ*_C_ = 25.9 (q); 27.5 (q); 28.0 (s); 32.3 (s); 38.9 (t); 51.8 (t); 58.9 (s); 112.6 (s); 117.3 (s); 123.0 (d); 149.2 (d); 152.7 (s); 154.2 (s); 158.4 (s); 198.9 (s) ppm. MS *m*/*z* (%): 309 (M^+^), 212 (100), 116, 83, 77, 56. Anal. Calcd for C_18_H_19_N_3_O_2_ (309.36): C, 69.88; H, 6.19; N, 13.58. Found: C, 69.85; H, 6.23; N, 13.55%.

**2-Amino-4-methyl-5-oxo-4-(pyridin-4-yl)-5,6,7,8-tetrahydro-4*H*-chromene-3-carbonitrile 4b:** Colorless crystals (EtOH), yield (2.39 g, 85%), mp. 223 °C. IR υ_max_ = 3324 & 3214 (NH_2_), 2192 (CN), 1682 (C=O) cm^−1^. ^1^H NMR: *δ*_H_ = 1.73 (s, 3H, CH_3_), 1.85–1.97 (m, 2H, CH_2_), 2.16–2.22 (m, 2H, CH_2_), 2.44–2.54 (m, 2H, CH_2_), 6.82 (s, 2H, D_2_O exchangeable, NH_2_), 7.22 (d, *J* = 5.1 Hz, 2H, pyridine-H_3_, H_5_), 8.55 (d, *J* = 5.1 Hz, 2H, pyridine-H_2_, H_6_) ppm. ^13^C NMR: *δ*_C_ = 25.92 (q); 21.15 (t); 28.0 (s); 29.3 (t); 37.05 (t); 58.9 (s); 112.62 (s); 117.31 (s); 123.05 (d); 149.2 (d); 152.72 (s); 154.2 (s); 158.41 (s); 198.8 (s) ppm. MS *m*/*z* (%): 281 (M^+^), 202, 173(100), 95, 77, 55. Anal. Calcd for C_16_H_15_N_3_O_2_ (281.31): C, 68.31; H, 5.37; N, 14.94. Found: C, 68.25; H, 5.33; N, 14.95%.

### 3.2 Synthesis of 2-(1-(pyridin-4-yl)ethylidene)malononitrile 5

To 4-acetylpyridine **1** (1.21g; 10 mmol) in 20 mL of dry EtOH, we added malononitrile **2** (0.66g, 10 mmol) and 2–3 drops of a freshly prepared solution of NaOEt in dry EtOH. The flask's contents started boiling vigorously. After leaving the mixture to cool overnight, a crystalline product formed, which we filtered out and washed multiple times with cold EtOH to obtain a pure compound **5**. Bright brown crystals (EtOH), yield (1.44 g, 85%), mp. 270 °C. IR υ_max_ = 2223 (CN) cm^−1^. ^1^H NMR: *δ*_H_ = 2.44 (s, 3H, CH_3_), 7.45 (d, *J* = 5.1 Hz, 2H, pyridine-H_3_, H_5_), 8.55 (d, *J* = 5.1 Hz, 2H, pyridine-H_2_, H_6_) ppm. ^13^C NMR: *δ*_C_ = 23.0 (q); 83.5 (s); 114.6 (s); 120.5 (d); 144.3 (s); 149.7 (d); 171.9 (s) ppm. Anal. Calcd for C_10_H_7_N_3_ (169.18): C, 70.99; H, 4.17; N, 24.84. Found: C, 71.05; H, 4.22; N, 24.75%.

### 3.3 Alternative synthesis I for compounds 4a and 4b

A mixture of 1.69g (10 mmol) of 2-(1-(pyridin-4-yl)ethylidene)malononitrile **5** with either of the β-diketones **3a** and **3b** (1.4g, 10 mmol of dimedone **3a**; or 1.12g, 10 mmol of cyclohexane-1,3-dione **3b**) in dry ethanol (20 mL) followed by 2–3 drops of freshly prepared NaOEt solution in dry EtOH, and the mixture of the flask boiled for 1h. After cooling the flask contents overnight, the resulting precipitated crystalline product in each instance was separated via filtration. It underwent multiple washes using cold EtOH and was subsequently recrystallized from EtOH to obtain analytically pure compounds. These compounds were found to be identical in terms of MP's, IR, and ^1^H NMR with **4a** and **4b**, respectively.

### 3.4 Condensation of 4-acetyl pyridine 1 with β-diketones 3a and 3b: synthesis of 8a and 8b

4-Acetylpyridine **1** (1.21g; 10 mmol) was combined with dimedone **3a** (1.4g, 10 mmol) or cyclohexane-1,3-dione **3b** (1.12g, 10 mmol) in dry EtOH (20 mL). Next, 2–3 drops of freshly prepared NaOEt solution in dry EtOH were added to the mixture, and the flask was boiled for 1 h. After cooling the flask contents overnight, a crystalline product precipitated in each case. The precipitates were filtered and washed several times with cold EtOH. Finally, the obtained products **8a** and **8b** were recrystallized from EtOH to ensure their purity.

### 3.5 5,5-Dimethyl-2-(1-pyridin-4-yl)ethylidene)cyclohexane-1,3-dione 8a

Yellow crystals (EtOH), yield (2.0 g, 83%), mp. 223 °C. IR υ_max_ = 1683 & 1690 (2C=O) cm^−1^. ^1^H NMR: *δ*_H_ = 1.0 (s, 6H, 2CH_3_), 2.25 (dd, 4H, CH_2_), 2.4 (s, 3H, CH_3_), 7.46 (d, *J* = 5.1 Hz, 2H, pyridine-H_3_, H_5_), 8.55 (d, *J* = 5.1 Hz, 2H, pyridine-H_2_, H_6_) ppm. ^13^C NMR: *δ*_C_ = 20.9 (q); 26.5 (q); 30.35 (s); 52.1 (t); 140.15 (s); 120.7 (d); 144.3 (s); 149.65 (d); 176.4 (s); 194.5 (s) ppm. Anal. Calcd for C_15_H_17_NO_2_ (243.31): C, 74.05; H, 7.04; N, 5.76. Found: C, 74.0; H, 7.09; N, 5.82%.

### 3.6 2-(1-Pyridin-4-yl)ethylidene)cyclohexane-1,3-dione 8b

Pale yellow crystals (EtOH), yield (1.76 g, 82%), mp. 227 °C. IR υ_max_ = 1682&1686 (2C=O) cm^−1^. ^1^ H NMR: *δ*_H_ = 1.53 (p, 2H, CH_2_), 3.16 (t, 4H, 2CH_2_), 2.43 (s, 3H, CH_3_), 7.46 (d, *J* = 5.1 Hz, 2H, pyridine-H_3_, H_5_), 8.55 (d, *J* = 5.1 Hz, 2H, pyridine-H_2_, H_6_) ppm. ^13^C NMR: *δ*_C_ = 15.12 (t); 20.8 (q); 39.15 (t); 120.75 (d); 140.15 (s), 144.3 (s), 149.55 (d); 176.41 (s); 194.5 (s) ppm. Anal. Calcd for C_13_H_13_NO_2_ (215.25): C, 72.54; H, 6.09; N, 6.51. Found: C, 72.50; H, 6.13; N, 6.54%.

### 3.7 Alternative synthesis II for compounds 4a and 4b

5,5-Dimethyl-2-(1-pyridin-4-yl)ethylidene)cyclohexane-1,3-dione **8a** (2.43g, 10 mmol) or 2-(1-pyridin-4-yl)ethylidene)cyclohexane-1,3-dione **8b** (2.15g, 10 mmol) were dissolved in 25 mL of absolute EtOH and heated until the substances were fully dissolved. To this clear solution, malononitrile **2** (0.66g, 10 mmol) was added, followed by 2–3 drops of freshly prepared NaOEt. The mixture was further heated for 1 h and left to cool overnight. The resulting precipitate was filtered, washed with cold EtOH, and then recrystallized from EtOH. The obtained products were confirmed to be identical to compounds **4a** and **4b** based on their melting points, infrared spectra, and ^1^H NMR spectra.

### 3.8 Docking study

**Ligand Preparation**: The molecular modeling for the synthesized compounds **4a, 4b, 5, 8a,** and **8b** was conducted using the Molecular Operating Environment software. Chemdraw 12.0 was utilized to sketch these compounds. All minimizations were performed until a root mean square deviation gradient of 0.1 kcal mol^−1^Å^−1^ using the MMFF94x (Merck Molecular Force Field 94x) method.

### 3.9 Protein preparation

Protein data was obtained from the RCSB database (www.rcsb.org), specifically PDB ID: 1SA0, which is the crystal structure of a tubulin complex with its GDP binding site, featuring a resolution of 3.58 Å [[67], [68], [69]]. The enzyme setup for docking experiments was carried out in alignment with standard methodologies. This setup process entailed several steps [70]: 1) Keeping only the GDP from among the small molecules; 2) Appending hydrogen atoms to the enzyme's structure to correct the geometry and mend any disrupted bonds; 3) Utilizing Alpha Site Finder to introduce dummy atoms into the large site of the enzyme structure [71]; 4) Storing the resulting pocket in Moe format for the analysis of potential ligand-enzyme interactions at the active docking site; 5) Evaluating the interactions of ligands with the amino acids at the active site. For docking, the Triangle Matcher method was employed for placement, alongside the London dG score for evaluating binding affinity, where a lower score signifies a stronger affinity between the molecule and the protein [36]. Post-docking, the two-dimensional and three-dimensional interactions with amino acid residues were examined. The procedures for docking and the results were systematically recorded [72].

## 4 Conclusions

The study successfully demonstrates a multi-component reaction (3MCR) using 4-acetylpyridine, malononitrile, and either dimedone or cyclohexane-1,3-dione, catalyzed by sodium ethoxide in ethanol to produce novel 4-methyl-4-(pyridine-4-yl)-5,6,7,8-tetrahydro-4*H*-chromene derivatives (compounds **4a** and **4b**). Detailed investigations into the reaction mechanism and structural confirmation of these compounds were carried out using analytical, spectral, and X-ray crystallography. Additionally, docking studies highlighted their potential interaction with tubulin, showing strong binding affinities, which points to their promising applications in pharmaceuticals and agrochemicals. Importantly, these compounds meet Lipinski's rule of five, indicating their suitability as drug candidates. This comprehensive study not only adds to the chemical synthesis literature but also opens up possibilities for their use in medicinal and agricultural products.

Crystallographic data (excluding structure factors) for the structure **4a** reported in this paper have been deposited with the Cambridge Crystallographic Data Centre as supplementary publication no. CCDC-2241780. Copies of the data can be obtained free of charge on application to CCDC, 12 Union Road, Cambridge CB2 1EZ, UK [fax.: (Internat.)] + 441223/336-033; e-mail: deposit@ccdc.cam.ac.uk].

## Data availability statement

The data presented in this study are available on request from corresponding author.

## CRediT authorship contribution statement

**Fathy M. Abdelrazek:** Writing – review & editing, Writing – original draft, Methodology, Formal analysis, Conceptualization. **Magdi E.A. Zaki:** Writing – review & editing, Writing – original draft, Investigation, Funding acquisition, Formal analysis. **Sami A. Al-Hussain:** Writing – review & editing, Writing – original draft, Investigation. **Basant Farag:** Writing – review & editing, Writing – original draft, Methodology. **Ali M. Hebishy:** Writing – review & editing, Supervision. **Mohamed S. Abdelfattah:** Writing – review & editing, Writing – original draft, Supervision. **Safaa M. Hassan:** Writing – review & editing, Methodology, Formal analysis, Data curation. **Ahmed F. El-Farargy:** Writing – review & editing, Methodology, Formal analysis. **Lyuba Iovkova:** Methodology, Software, Writing – original draft, Writing – review & editing. **David Mross:** Methodology, Visualization, Writing – original draft, Writing – review & editing. **Sobhi M. Gomha:** Formal analysis, Methodology, Supervision, Writing – review & editing.

## Declaration of competing interest

The authors declare that they have no known competing financial interests or personal relationships that could have appeared to influence the work reported in this paper.
